# Supplementary material for: A Membrane-Assisted Mechanism for the Release of Ceramide from the CERT START Domain
Source: J Phys Chem B. 2024 Jun 21;128(26):6338–51. doi: 10.1021/acs.jpcb.4c02398 (PMC11228987; doi:10.1021/acs.jpcb.4c02398)
Supplement: Supplementary file 1 — jp4c02398_si_001.pdf [file jp4c02398_si_001.pdf]

Supplementary Materials for

A Membrane-assisted Mechanism for the Release of Ceramide from the CERT START Domain

Mahmoud Moqadam[1,2], Parveen Gartan[1,2], Reza Talandashti[1,2], Antonella Chiapparino[3], Kevin Titeca[3,4], Anne-Claude Gavin[4], Nathalie Reuter[1,2*]

[1] Department of Chemistry, University of Bergen, Bergen 5020, Norway
[2] Computational Biology Unit, Department of Informatics, University of Bergen, Bergen 5020, Norway
[3] European Molecular Biology Laboratory, EMBL, Meyerhofstrasse 1, D-69117 Heidelberg, Germany
[4] University of Geneva, Department of Cell Physiology and Metabolism, CMU Rue Michel-Servet 1 1211 Genève 4, Switzerland

* Corresponding author. Email: nathalie.reuter@uib.no

Table of Contents:

1) Supplementary Text S1-S2 .... S2-S4
2) Figures S1-S16 .... S5-S20
3) Tables S1-S8 .... S21-S31
4) Movies S1-S4 .... S32
5) References S33-S34

Supplementary Text.

S1. Free energy calculations, simulation protocols

1. System preparation.

START apo and START-Cer. The structure of the START-Cer complex was retrieved from the protein data bank (PDB ID: 2e3q) and processed with MMTSB[1] tools and CHARMM-GUI. The CHARMM simulation package[2] (v47a2) was then used with the CHARMM36 force field for proteins and lipids to generate the topology (psf) and coordinates (crd) files for the complex. The complex was energy-minimized (steepest descents: 100 steps, adopted basis Newton-Raphson: 1000 steps), solvated in a cubic box (a=b=c= 86.1 Å) with TIP3P water molecules (edge cutoff=10 Å) and neutralized by adding 3 counter K^+ ions, replacing 3 randomly chosen water molecules from the bulk. This was followed by energy-minimization (steepest descent: 200 steps, adopted basis Newton-Raphson: 1000 steps). Particle mesh Ewald (PME) was used for long-range electrostatic interactions and periodic boundary conditions with a non-bonded cutoff of 16 Å along with truncation (vswitch) of van der Waals interactions at 10 Å. The system was then gradually heated from 190 K to 310 K with increments of 1 K every 100 steps using a gaussian distribution for velocity assignments. SHAKE[3] was used to constrain bonds between heavy atoms and hydrogen atoms. A short 5 ns equilibration and then 400 ns production simulations were run in the NPT ensemble with a 2-fs integration time step and using BLaDE[4] in CHARMM 47a2. The temperature was set to 310 K and controlled using a Langevin thermostat (drag coefficient = 0.1 ps^{-1}). A Monte Carlo barostat was used (target pressure=1 atm) with pressure coupling moves every 25 steps and the default (100 $Å^3$) standard deviation of the gaussian distribution was used for drawing volume changes.
The trajectory obtained was recentered around the START domain using CHARMM v.47a2 and clustered, using ttclust[5] and the RMSD of the domain backbone atoms. A structure from the largest cluster was selected for free energy calculations. Cer was removed from that structure to generate the structure of apo START (except for the N504 mutations for which the cluster closer to the X-ray structure was used).

START-Cer-POPC and START-POPC. The structure of the START-Cer-POPC complex was taken from the holo-Neu_r1 simulation. The conformation was chosen such that the Cer and POPC tails were present and before Cer leaves the binding site (t=886.9 ns). The START-POPC structure was obtained from the above by removing Cer.

2. Multisite lambda dynamics (MSλD)

The apo START structure and the START-Cer complex structure extracted from the cluster above were solvated (cutoff 15 Å) in a cubic box with TIP3 water molecules and neutralized by adding three K+ ions using the MMTSB toolset. These systems are denoted as **START-Water** and **START-Cer-Water** respectively in Table S5. The structures extracted from the holo-Neu r1 simulation were also similarly solvated and neutralized and are referred as **START-Cer-POPC-Water** and **START-POPC-water** in Table S6.
The mutant atoms on the perturbation site were added using the PATCH command in the CHARMM program[6]. The perturbation site had all the atoms of the wild-type

residue and all the atoms for the mutant (including backbone).[7] All valence angles, dihedrals and impropers were removed between the alchemical groups. The alchemical system containing two substituents on a single site was set up using the BLOCK module[7] of CHARMM and atoms of both substituents were constrained using the SCAT (force constant = 118.4) facility. A FNEX value of 5.5 was used for the functional form of λ. Soft core potentials were used along with the ALF[8] biases for each of the substituents. The POPC tail outside the START cavity was harmonically restrained with a force constant of 2 kcal*mol^{-1}*Å^{-2} on heavy atoms.
The START-Water and START-Cer-Water systems were simulated using BLaDE with λ values saved every 20 fs and a non-bonded cutoff of 12 Å along with truncation (vswitch) of van der Waals interactions between 9-10 Å while keeping the remaining conditions and parameters same as for the equilibrium simulations described earlier in this section (Cf *System preparation)*. The START-Cer-POPC-Water and START-POPC-Water systems were simulated with DOMDEC[9] since BLaDE does not support harmonic restraints. In these simulations the Nosé-Hoover thermostat (temp = 310 K; tmass = 1000 kcal*ps^2) was used for temperature control and a Langevin piston (friction coefficient: 20 ps^{-1}) for pressure control. All the simulations either with BLaDE or DOMDEC were performed with CHARMM 47a2.
MSλD simulations were performed in an iterative fashion.[10,11] The first phase consisted of 50-60 small 100 ps long simulations to get an idea regarding the biases and, the second phase consisted of 10-20 longer 1 ns simulation to flatten the biases. The ALF biases were calculated using weighted histogram analysis method (WHAM)[12] and optimized after every run. The production runs were started with the lambda landscape flattened in 5 independent replicas of 5 ns each. The final relative free energy differences are obtained via the following equation:

$$\Delta\Delta G = -k_B T ln(\frac{P(\lambda_j = 1)}{P(\lambda_i = 1}) - (\phi_j - \phi_i) \quad (1)$$

Where, i and j are the wild-type and mutant residues respectively.
P – probability
ϕ – bias free energy

S2. Replicate MD Simulations

We conducted two replicates of the simulations for each START-lipid bilayers systems (see Table S1); the results of the first replicates are presented in the main text. In this section, we report our observations from the second replicates. Our findings suggest that only POPC can insert a tail into the cavity, positioning its head group between the Ω1 and Ω4 loops.

Apo-ER_r2. A POPE lipid occupied the site between Ω1 and Ω4, and the cavity remained closed throughout the simulation. No gate opening was observed during the simulation.

Apo-neutral_r1. a POPC lipid occupied the site between Ω1 and Ω4 snorkeling to insert a tail in the open cavity. Simultaneously another POPC lipid blocked the

entrance into the cavity by placing its headgroup near the cavity. No tail insertion was observed during the simulation.

Holo_neutral_r2 / holo-Golgi_r2. we also observed the opening of the cavity and lipid snorkeling events. In both simulations, a POPC occupied the site between Ω1 and Ω4 loops and snorkels around the entrance to the cavity, but neither tail insertion nor ceramide release were observed.

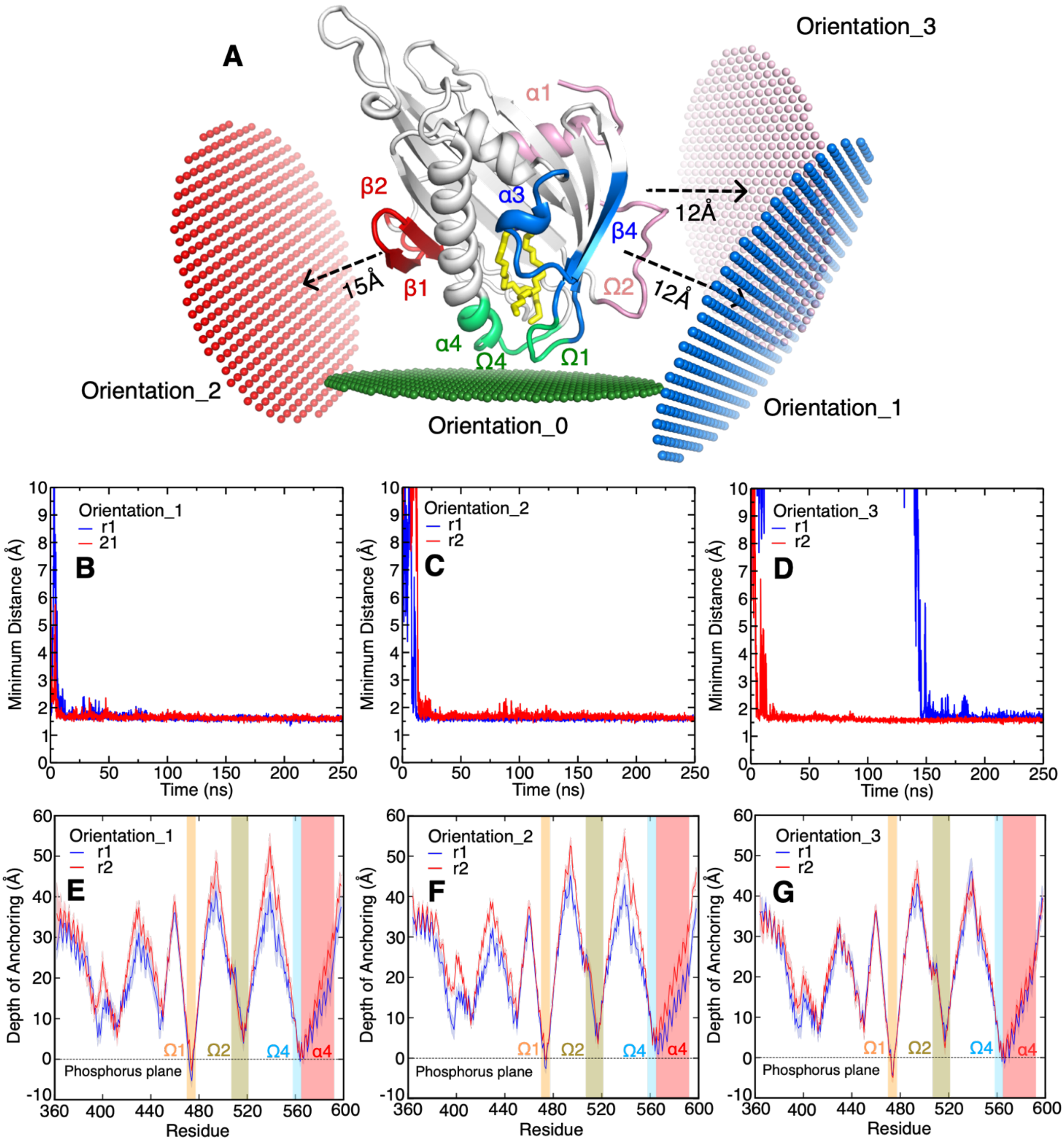

Fig. S1. Alternative starting orientations of START with respect to bilayers. (**A**) Numbers under the arrows report the initial minimum distance between START and bilayers. All simulations (including the replicates) were run for 250 ns and lead to the START domain anchored at the bilayer through the C-terminus α4 helix, the Ω1, Ω2 and Ω4 loops. The Orientation_0 corresponds to the starting orientation used in the main text (See also Fig 2 in main text). (**B-D**) Minimum distance between START and bilayers through the simulations. (**E-G**) Depth of insertion of each START amino acids (Cα of glycine and Cβ of others) into the bilayers for both replicas (r1 and r2) in each case, calculated using the last 50 ns of each simulation.

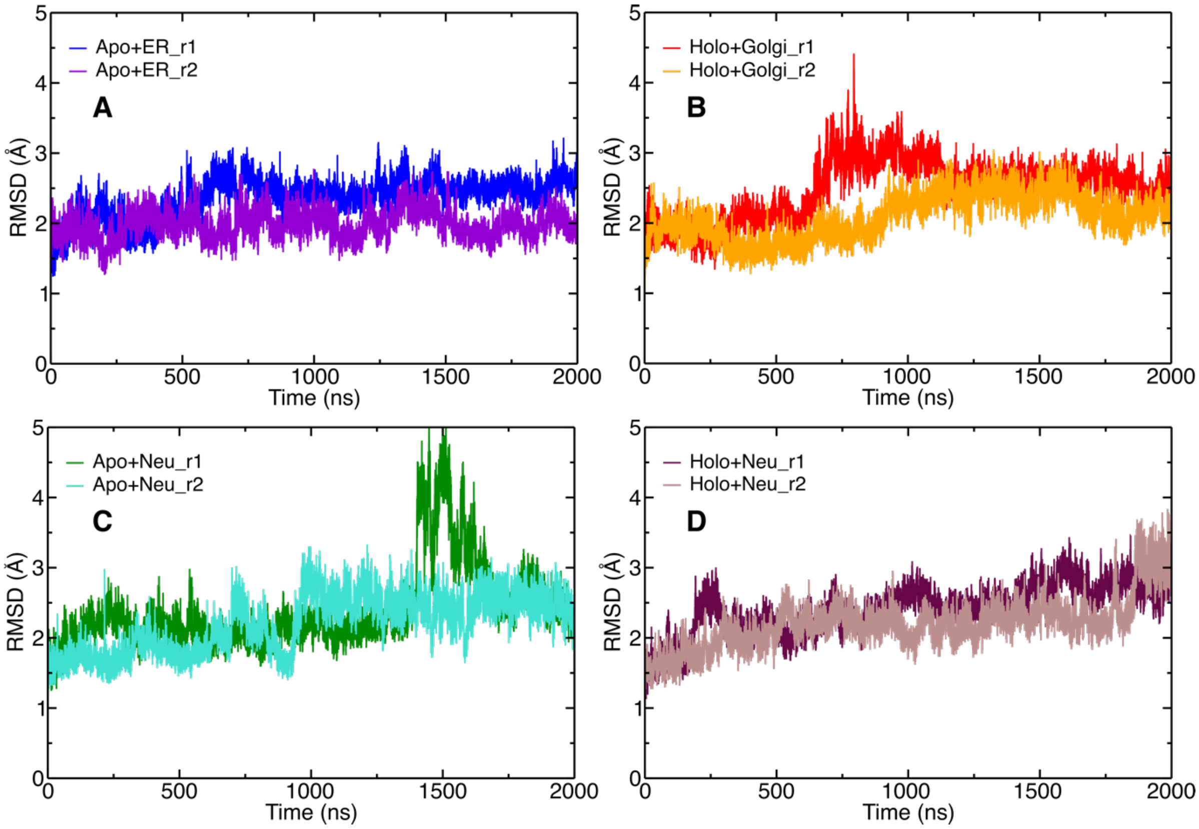

Fig. S2. RMSD plots. RMSD for both replicas (r1 and r2) in each case, calculated for START domain excluding the N terminus helix, resid 362-391. (**A**) Apo in the ER-like bilayer (**B**) Holo in the Golgi-like bilayer (**C**) Apo in the neutral bilayer (**D**) Holo in the neutral bilayer.

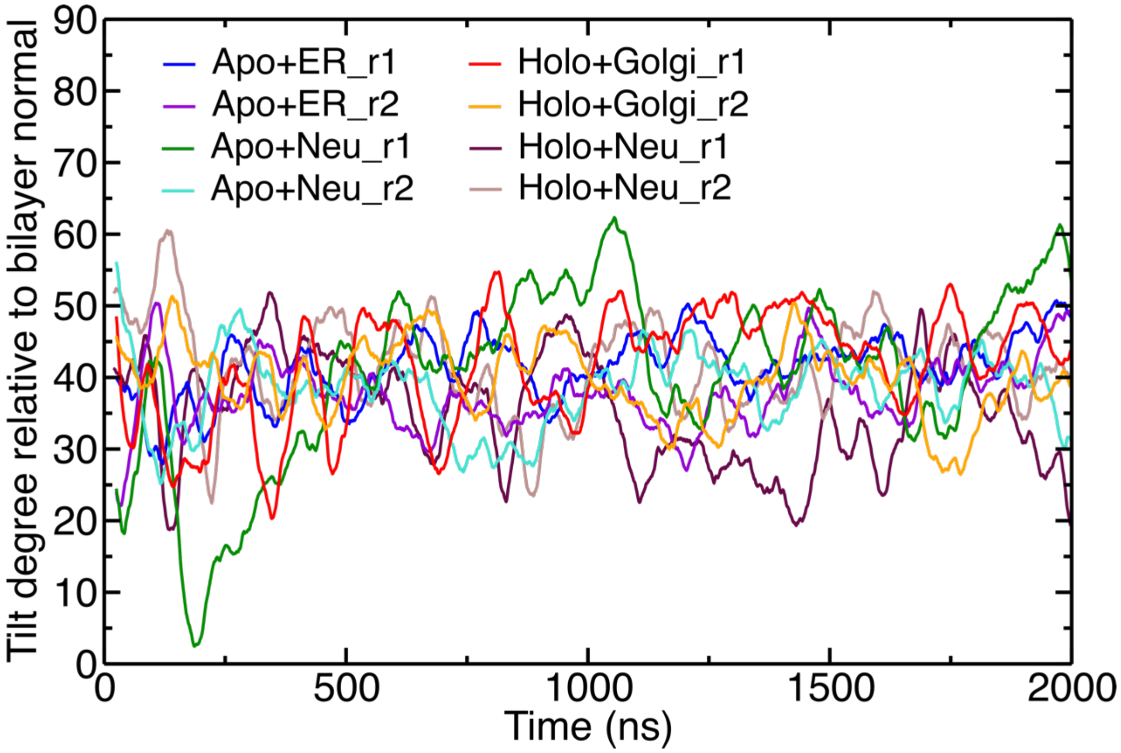

Fig. S3. Tilt angle of apo and holo forms on the different bilayers. The tilt is defined using the START domain α4 vector with respect to the bilayer normal.

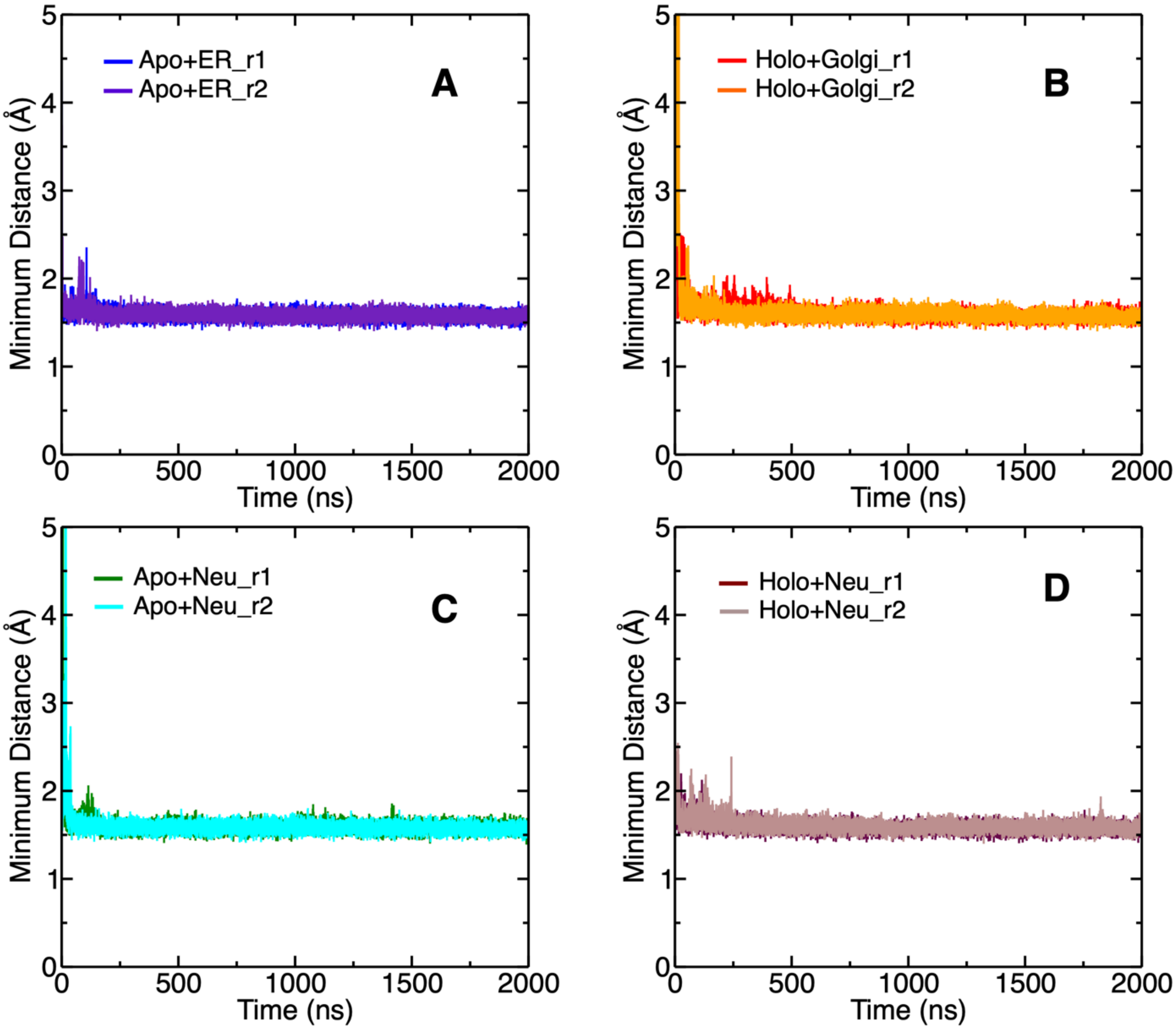

Fig. S4. START domain and bilayer distance. Minimum distance between the START domain and the bilayer along the 2 μs simulations: (**A**) apo and ER bilayer, (**B**) holo and Golgi bilayer, (**C**) apo and neutral bilayer and (**D**) holo and neutral.

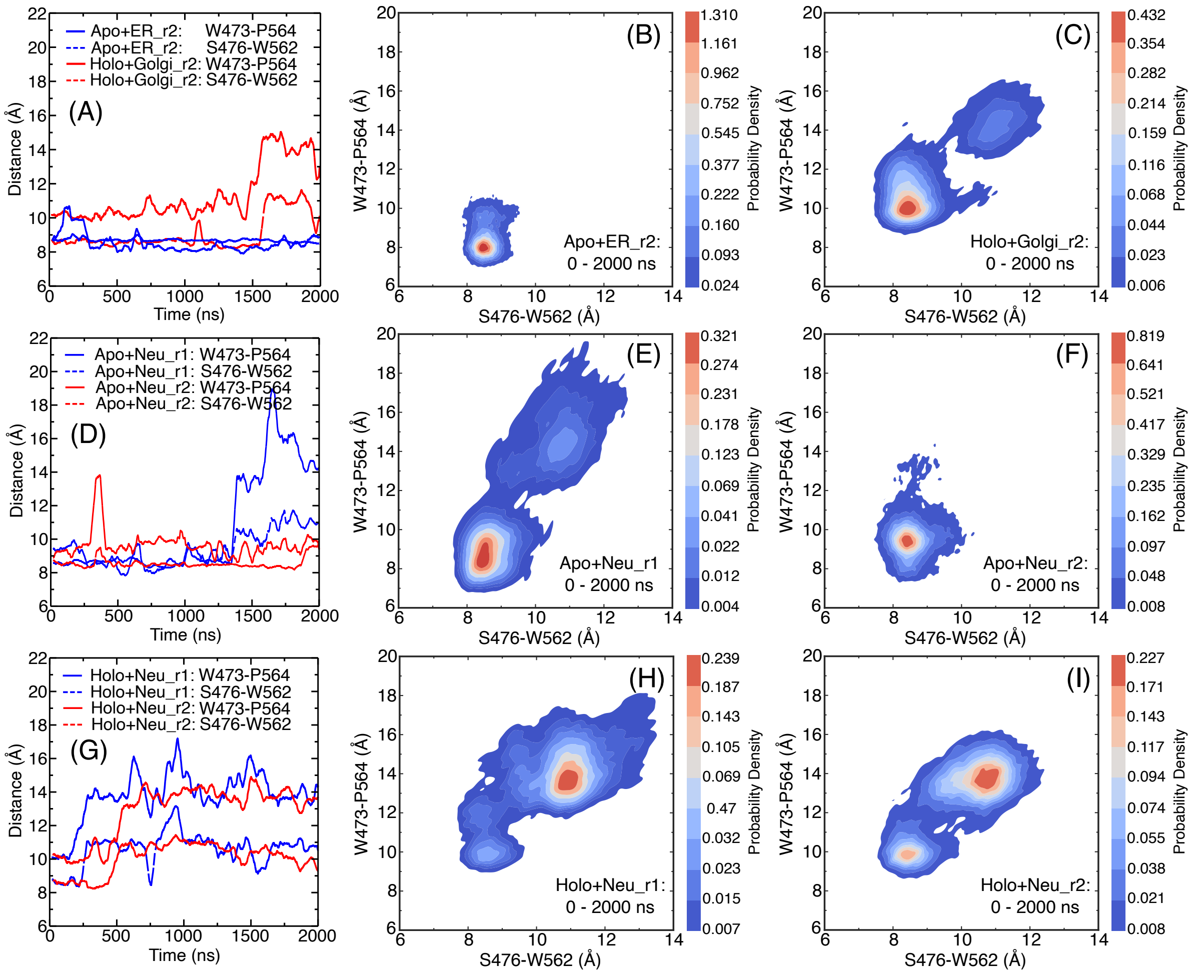

Fig. S5. Gate opening through displacements of Ω1, Ω4 and α4. (**A, D, and G**) Time series of distances between W473 (Ω1) and P564 (Ω4) (plain lines), and S476 (Ω1) and W564 (Ω4) (dotted lines) in simulations of START with the ER (**A**), Golgi (**A**), and neutral (**D, G**) bilayers. (**B, C, E, F, H, and I**) Estimated probability density (KDE function) of the closed and open states of START calculated from the simulation trajectories with the ER bilayer (**B**), the Golgi bilayer (**C**), the neutral bilayer (Neu) (**E, F, H, and I**). The KDE (Kernel density estimation) represents the data using a continuous probability density curve. The bar at the right side shows the intensity of data values along the KDE curve.

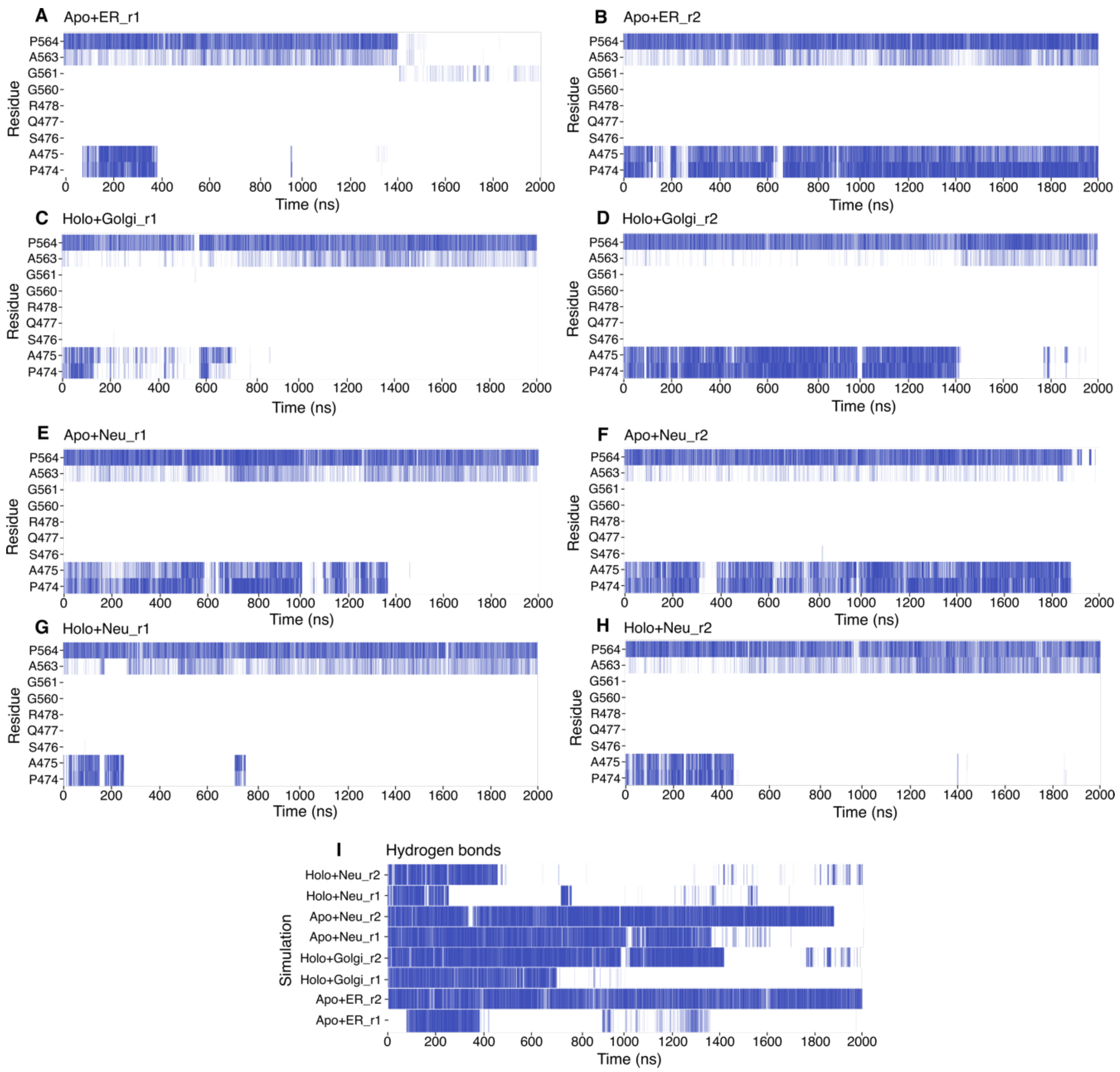

Fig. S6. Hydrophobic contacts and hydrogen bonds. Time series of hydrogen bonds and hydrophobic contacts between W562 and its nearest neighbors in the Ω1 and Ω4 loops. (**A-H**) Hydrophobic contacts (**I**) Hydrogen bonds. The plots display a bar if there is at least one hydrophobic contact (A-H) or hydrogen bond (**I**).

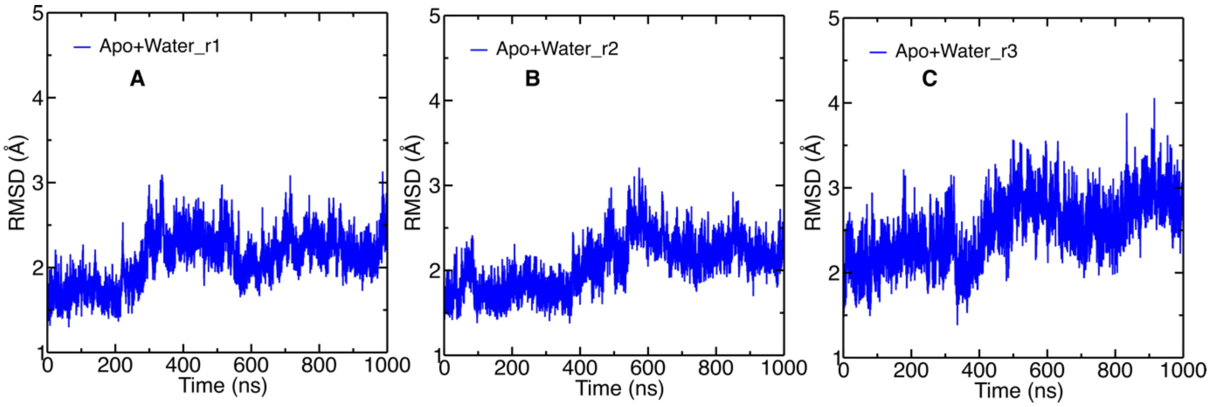

Fig. S7. Backbone RMSD for apo START in water in three replicate simulations. The N-terminus helix (residues number 362-391) is not included.

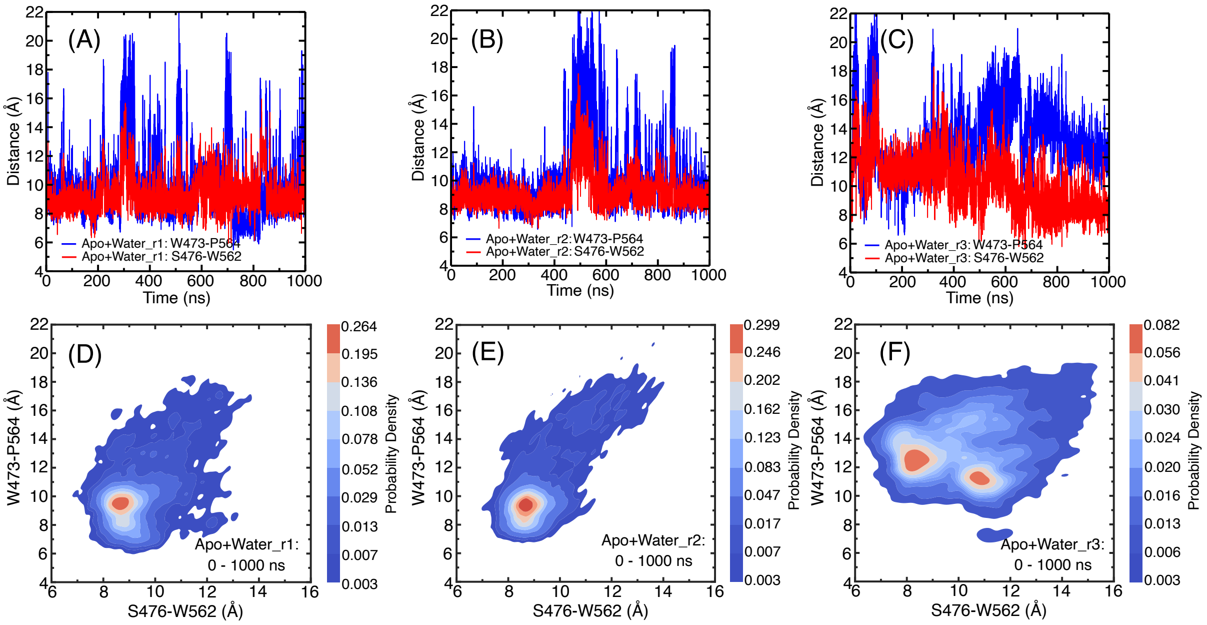

Fig. S8. Gate opening through displacements of Ω1, Ω4 and α4. (**A-C**) Time series of the W473(Ω1)-P564(Ω4) in blue and S476(Ω1)-W562(Ω4) in red distances in each of the three replicate simulations of apo START in water. (**D-F**) Density distributions (KDE function) of the distances plotted in A-C and characterizing the closed and open states of START. The KDE (Kernel density estimation) represents the data using a continuous probability density curve. The bar at the right side shows the intensity of data values along the KDE curve.

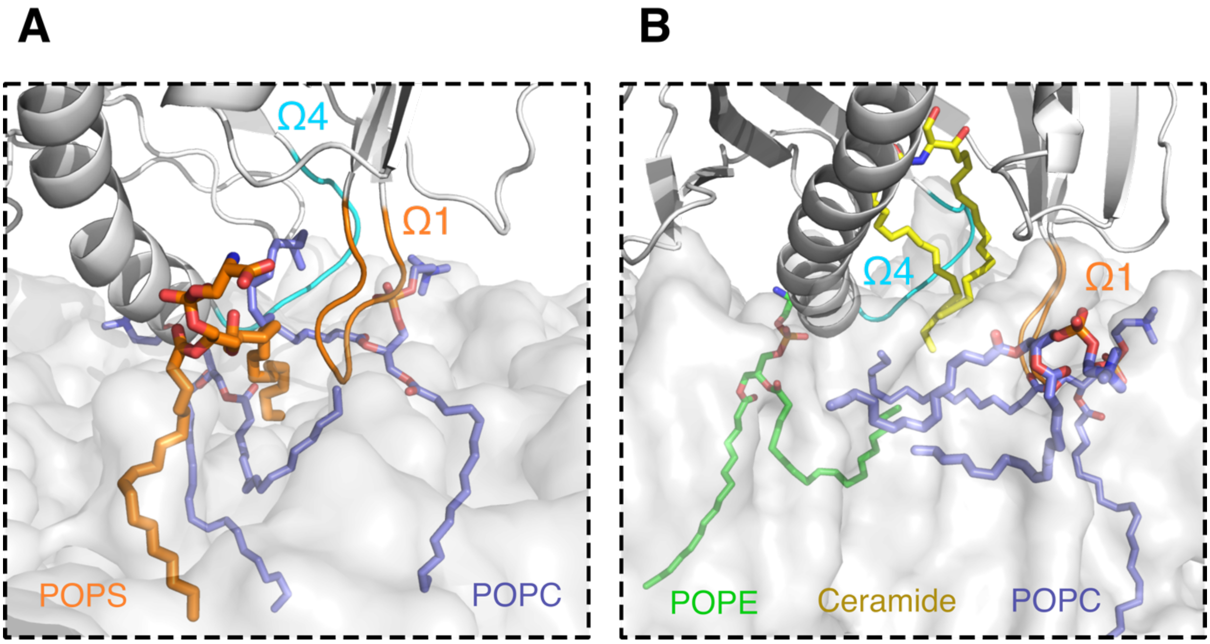

Fig. S9. Lipid Snorkeling. Snapshots illustrating lipid snorkeling under the START domain in (**A**) the ER-like and (**B**) the Golgi-like bilayers.

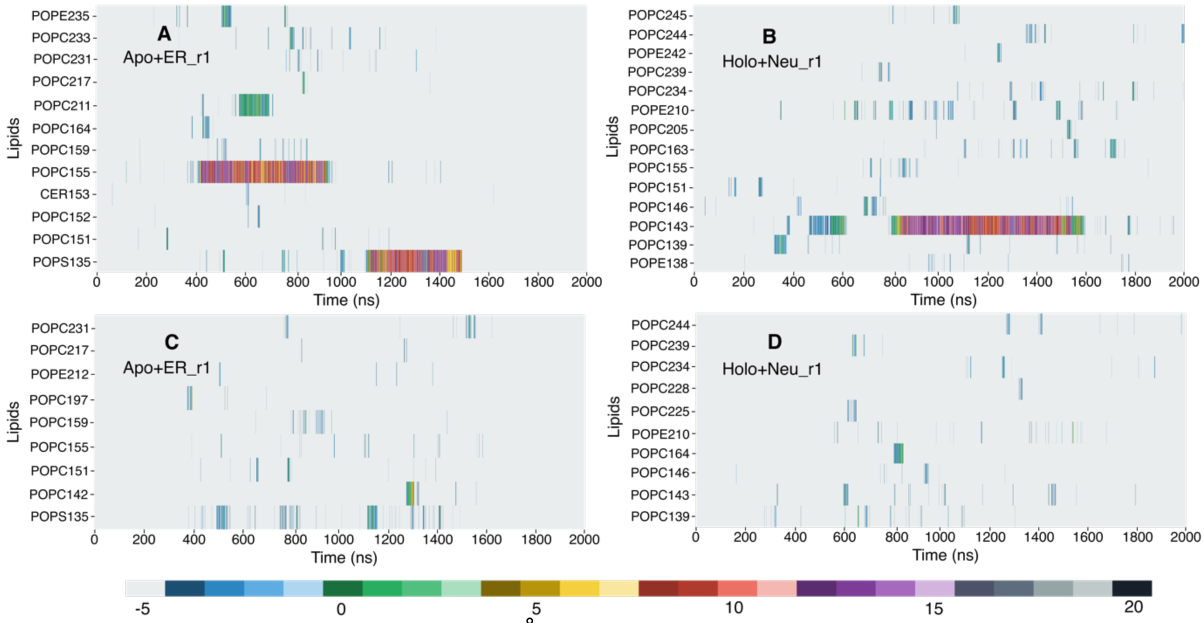

Fig. S10. Lipid snorkeling within 3Å of the START domain in the ER and neutral simulations. Z value of the C218 of the POPX (X: C, E, I, and S) and the C18S atoms of the ceramide with respect to the average plane of the phosphorus atoms (Z=0) (**A**) apo+ER_r1 (**B**) holo+Neu_r1. Z value of the C316 of the POPX (X: C, E, I, and S) and the C18F atoms of the ceramide with respect to the average plane of the phosphorus atoms (Z=0) (**C**) apo+ER_r1 (**D**) holo+Neu_r1.

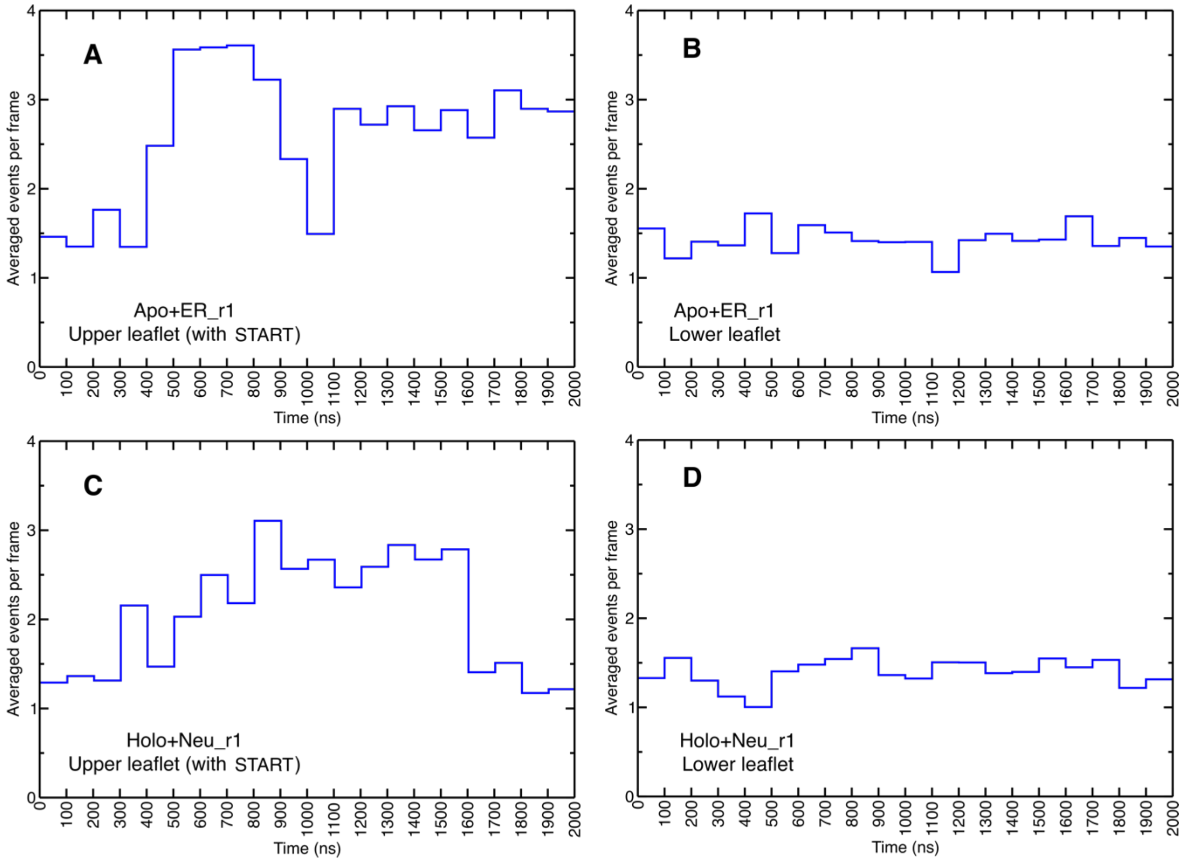

Fig. S11. Average number of snorkeling events. Number of times the C218 and C316 atoms of the POPX (X: C, E, I, and S) and the C18S and C18F atoms of the ceramide are at least 5 Å higher than the average plane of the phosphorus atoms. The numbers are average per frame in windows of 100 ns each. Data shown for lipids from (**A**) the upper leaflet (to which the START domain is bound) and (**B**) the lower leaflet in the apo+ER_r1 simulation; and for lipids in (**C**) the upper leaflet (with the START domain) and (**D**) the lower leaflet in the holo+Neu_r1 simulation. The lower leaflets are shown for reference.

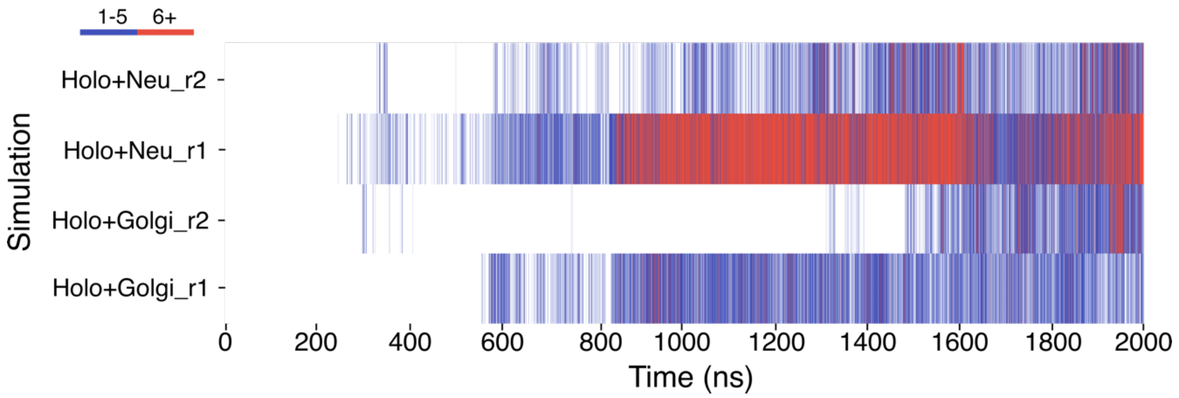

Fig. S12. Hydrophobic contacts between bound ceramide and lipid bilayer. Number of hydrophobic contacts between the START-bound ceramide and the lipids of the bilayer in the holo+Golgi and Holo+Neu simulations. Color bar indicates the number of hydrophobic contacts in each frame; Blue: 1-5 hydrophobic contacts, red: 6 or more hydrophobic contacts.

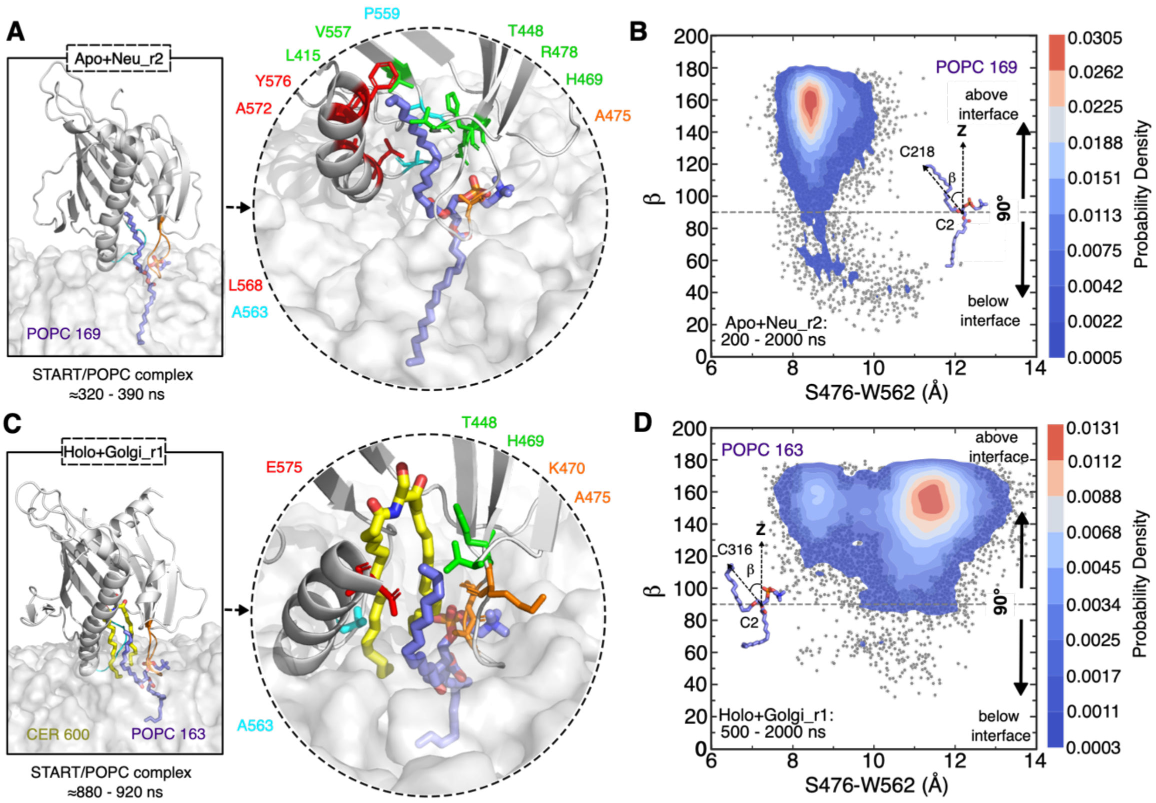

Fig S13. POPC (1-palmitoyl-2-oleoyl-phosphatidylcholine) rearrangement induced by START domain in opened state detected by MD simulation. (**A**) Close-up view of the binding conformation of POPC within apo START domain, and the amino acids in the cavity involved in hydrophobic contacts with the POPC tail in the neutral bilayer. (**B**) Distribution of the POPC tail angle with respect to membrane normal in the neutral bilayer and their estimated probability density (KDE function) are presented. (**C**) Close-up view of the binding conformation of POPC within START-Cer complex, and the amino acids in the cavity involved in hydrophobic contacts with the POPC tail in the Golgi bilayer. (**D**) Distribution of the POPC tail angle with respect to membrane normal in the Golgi bilayer and their estimated probability density (KDE function) are presented. The KDE (Kernel density estimation) represents the data using a continuous probability density curve. The bar at the right side shows the intensity of data values along the KDE curve. The times at which the POPC tail inserts into and exits the cavity are given below the snapshots. Ceramide colored yellow, POPC colored purple, Ω1 colored orange and Ω4 colored cyan. START domain is shown as cartoon and colored grey. Membrane is shown as grey transparent surface. The gray dots represent all the sampled tilt angles.

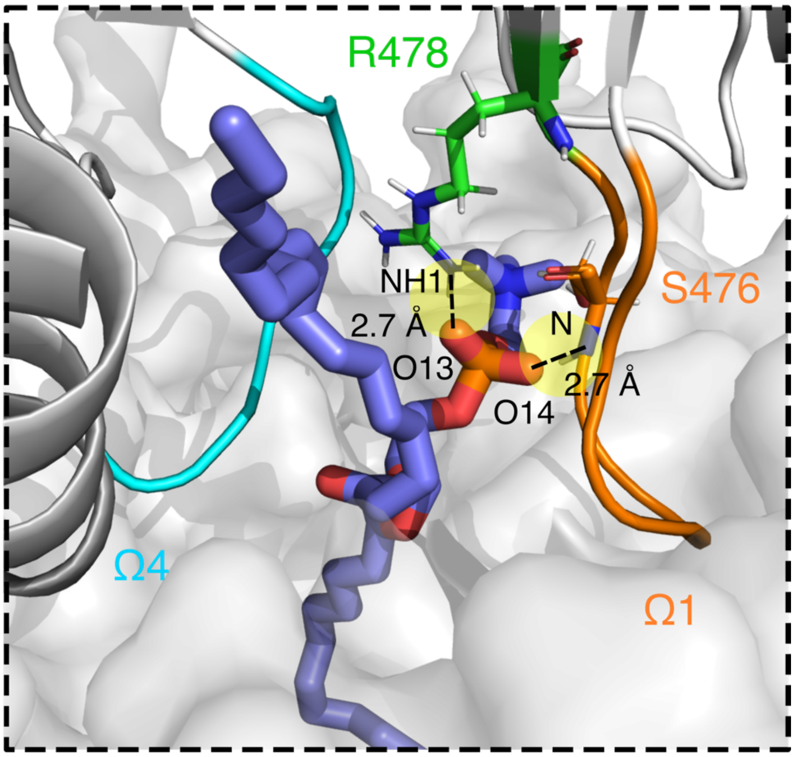

Fig. S14. The POPC lipid located between Ω1 and Ω4. The POPC head group is locked in place by a salt bridge between its phosphate group and R478, and by hydrogen bonds between the S476 side chain and the POPC phosphate group.

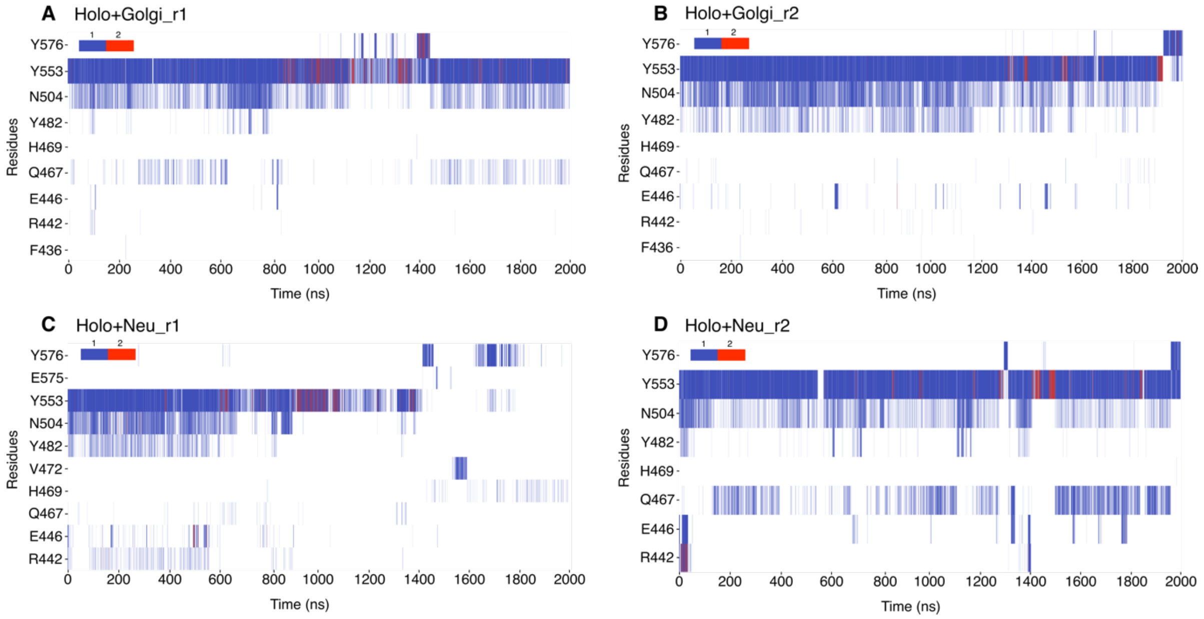

Fig. S15. Hydrogen bonds network around the ceramide head group. Time series along the Holo+Golgi_r1 (**A**), Holo+Golgi_r2 (**B**), Holo+Neu_r1 (**C**) and Holo+Neu_r2 (**D**) simulations. Color bar indicates the number of hydrogen bonds in each frame; Red: 2 hydrogen bonds, blue: 1 hydrogen bond.

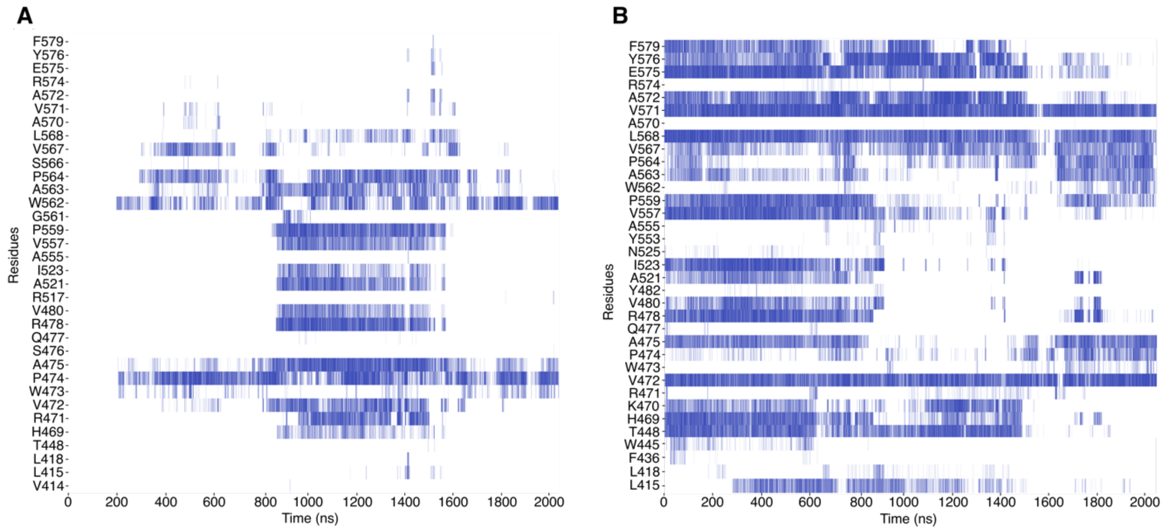

Fig. S16. All hydrophobic contacts in the holo+Neu_r1 simulation. Time series of contacts between (**A**) POPC143 and the START domain, between (**B**) ceramide and the START domain.

Table S1. Composition and size of the simulated systems. “r” stands for replicate. See Material and Methods for detailed simulation protocols.

#	Name	Domain	Lipid bilayer	Total number of atoms	Number of water molecules	Ions	Initial cell dimensions	SA/Lipid (Å^2)	Binding at (ns)
1	Apo+Water_r1	Apo	-	66858	21023	3 K^+	89.0<br>89.0<br>89.0	-	-
2	Apo+Water_r2								
3	Apo+Water_r3								
4	Apo+ER_r1	Apo	53% POPC<br>23% POPE<br>10% POPI<br>5% POPS<br>5% CHL<br>4% Cer	135953	33110	43 K^+	90.3<br>90.3<br>176.3	59.6	300
5	Apo+ER_r2			168992	44123		90.3<br>90.3<br>218.3	59.6	500
6	Apo+Neu_r1	Apo	60% POPC<br>30% POPE<br>5% CHL<br>4% Cer	147012	36931	3 K^+	89.9<br>89.9<br>191.3	58.4	400
7	Apo+Neu_r2			169467	44416		89.9<br>89.9<br>220.3	58.4	200
8	Holo+Neu_r1	Holo	60% POPC<br>30% POPE<br>5% CHL<br>4% Cer	143500	35731		89.9<br>89.9<br>186.9	58.5	200
9	Holo+Neu_r2			143500	35731		89.9<br>89.9<br>161.9	58.5	300
10	Holo+Golgi_r1	Holo	46% POPC<br>18% POPE<br>9% POPI<br>6% POPS<br>5% POPA<br>5% DAG<br>5% CHL<br>6% Cer	123852	29203	51 K^+	89.9<br>89.9<br>161.9	58.8	500
11	Holo+Golgi_r2			123852	29203		89.9<br>89.9<br>186.9	58.8	300
12	Holo+Golgi +POPC_r1	Holo + POPC		142813	35471		91.0<br>91.0<br>176.3	59.0	bound structure
13	Holo+Golgi +POPC_r2			142813	35471		91.0<br>91.0<br>176.3	59.0	bound structure
14	Orientation_1_r1	Apo	Same as #4 & #5 (ER)	159785	41054	43 K^+	90.3<br>90.3<br>206.1	59.5	100
15	Orientation_1_r2							59.5	100
16	Orientation_2_r1			156504	39968		90.3<br>90.3<br>202.6	59.5	100
17	Orientation_2_r2							59.5	150
18	Orientation_3_r1	Holo	Same as #10 & #13 (Golgi)	159330	41029	51 K^+	89.9<br>89.9<br>207.1	58.9	100
19	Orientation_3_r2							58.9	200

Table S2. Candidate atoms for the hydrophobic interaction analysis. The atom names correspond to the nomenclature of the CHARMM36 force field.

Residue/lipid	Candidate atoms
**ALA**	CA HA CB HB1 HB2 HB3
**ARG**	CA HA CB HB1 HB2 CG HG1 HG2
**ASN**	CA HA CB HB1 HB2
**ASP**	CA HA CB HB1 HB2
**CYS**	CA HA CB HB1 HB2
**GLN**	CA HA CB HB1 HB2 CG HG1 HG2
**GLU**	CA HA CB HB1 HB2 CG HG1 HG2
**GLY**	CA HA1 HA2
**HSD**	CA HA CB HB1 HB2
**HSE**	CA HA CB HB1 HB2
**HSP**	CA HA CB HB1 HB2 CG
**ILE**	CA HA CB HB CG1 HG11 HG12 CG2 HG21 HG22 HG23 CD HD1 HD2 HD3
**LEU**	CA HA CB HB1 HB2 CG HG CD1 HD11 HD12 HD13 CD2 HD21 HD22 HD23
**LYS**	CA HA CB HB1 HB2 CG HG1 HG2 CD HD1 HD2 CE HE1 HE2
**MET**	CA HA CB HB1 HB2 CG HG1 HG2 CE HE1 HE2 HE3
**PHE**	CA HA CB HB1 HB2 CG CD1 HD1 CD2 HD2 CE1 HE1 CE2 HE2 CZ HZ
**PRO**	CA HA CB HB1 HB2 CD HD1 HD2 CG HG1 HG2
**SER**	CA HA CB HB1 HB2
**THR**	CA HA CB HB CG2 HG21 HG22 HG23
**TRP**	CA HA CB HB1 HB2 CG CD1 HD1 CD2 CE3 HE3 CZ3 HZ3 CH2 HH2 CZ2 HZ2
**TYR**	CA HA CB HB1 HB2 CG CD1 HD1 CD2 HD2 CE1 HE1
**VAL**	CA HA CB HB CG1 HG11 HG12 HG13 CG2 FG21 HG22 HG23
**POPC/POPE/ POPI/POPS/ POPA/POGL**	C23 H3R H3S C24 H4R H4S C25 H5R H5S C26 H6R H6S C27 H7R H7S C28 H8R H8S C29 H9S1 C210 H10S1 C211 H11R H11S C212 H12R H12S C213 H13R H13S C214 H14R H14S C215 H15R H15S C216 H16R H16S C217 H17R H17S C218 H18R H18S H18T C33 H3X H3Y C34 H4X H4Y C35 H5X H5Y C36 H6X H6Y C37 H7X H7Y C38 H8X H8Y C39 H9X H9Y C310 H10X H10Y C311 H11X H11Y C312 H12X H12Y C313 H13X H13Y C314 H14X H14Y C315 H15X H15Y C316 H16X H16Y H16Z
**CER180**	C5S C6S H6S H6T C7S H7S H7T C8S H8S H8T C9S H9S H9T C10S H10S H10T C11S H11S H11T C12S H12S H12T C13S H13S H13T C14S H14S H14T C15S H15S H15T C16S H16S H16T C17S H17S H17T C18S H18S H18T H18U C2F H2F H2G C3F H3F H3G C4F H4F H4G C5F H5F H5G C6F H6F H6G C7F H7F H7G C8F H8F H8G C9F H9F H9G C10F H10F H10G C11F H11F H11G C12F H12F H12G C13F H13F H13G C14F H14F H14G C15F H15F H15G C16F H16F H16G C17F H17F H17G C18F H18F H18G H18H
**CHOL**	C4 H4A H4B C5 C6 H6 C7 H7A H7B C8 H8 C14 H14 C15 H15A H15B C16 H16A H16B C17 H17 C13 C18 H18A H18B H18C C12 H12A H12B C11 H11A H11B C9 H9 C10 C19 H19A H19B H19C C1 H1A H1B C2 H2A H2B C20 H20 C21 H21A H21B H21C C22 H22A H22B C23 H23A H23B C24 H24A H24B C25 H25 C26 H26A H26B H26C C27 H27A H27B H27C

Table S3: Inventory of interactions between START and the bilayer lipids. The data presented in the table are averages over two replicas in each case, calculated from START binding to the bilayers to the end of the simulations. SSE: Secondary structure elements. AA: amino acid, α: helix, Ω: loop, β: beta-strand, occ: occupancy. Average number of hydrophobic contacts per frame are reported if > 0.5. Hydrogen bond occupancies are reported if > 30%. All reported hydrogen bonds are formed between protein residues and lipid phosphate groups except W473 that forms hydrogen bonds with glycerol (marked with *). Cation-π interactions are reported regardless of the occupancy.

SSE	AA	APO		HOLO	
		ER	Neu	Golgi	Neu
**Hydrophobic contacts (avg. per frame)**					
Ω1	V472	1.8	1.0	1.0	1.6
	W473	5.5	5.4	4.9	5.2
	P474	4.0	3.4	3.0	4.0
Ω4	W562	2.0	2.2	2.4	2.8
	P564	1.3	1.0	1.3	1.9
α4	S566	1.1	1.3	1.0	1.1
	V567	3.6	3.0	2.2	3.0
	V571	1.8	1.5	1.4	1.6
	R574	0.6	0.7	0.6	0.7
**Hydrogen bonds (%)**					
Ω1	R471	90.7	86.7	90.9	93.0
	W473	65.5*	74.2*	74.5*	68.2*
	S476	87.8	76.6	80.5	87.8
β6	R478	81.8	50.7	47.7	76.0
Ω2	R517	82.5	66.8	88.0	76.2
Ω4	W562	71.9	77.0	59.8	51.3
α4	A565	49.7	56.6	61.4	59.5
	S566	84.3	87.1	49.3	69.9
	R569	31.0	76.4	55.9	57.3
	K573	42.6	52.3	52.0	41.6
	R574	84.8	91.3	89.8	88.7
	K578	46.2	53.5	59.6	48.7
**Cation-π interactions (%)**					
Ω1	W473	11.3	5.5	1.1	3.6
Ω4	W562	19.5	17.7	20.2	13.4

Table S4. Occupancies (%) of lipid-tryptophane (W473, W562) hydrogen bonds. Hydrogen bonds between the two exposed tryptophanes and the lipid bilayers, reported in percentage of simulation time.

Simulations	W473			W562		
	Phosphate	Glycerol	Choline	Phosphate	Glycerol	Choline
Apo+ER_r1	22.9	71.9	5.2	87.7	8.2	4.1
Apo+ER_r2	39.1	48.0	12.9	90.5	7.3	2.2
Holo+Golgi_r1	33.7	62.7	3.6	88.0	11.3	0.7
Holo+Golgi_r2	32.1	56.7	1.0	65.1	22.5	3.2
Apo+Neu_r1	34.0	65.5	0.5	69.6	13.3	17.1
Apo+Neu_r2	14.3	85.6	0.1	83.8	16.1	0.1
Holo+Neu_r1	15.0	84.5	0.5	16.1	83.6	0.3
Holo+Neu_r2	42.9	54.8	2.3	93.5	6.4	0.1

Table S5. Occupancies (%) of Cer-START hydrogen bonds. Inventory of hydrogen bonds between ceramide and residues of the START domain cavity in simulations of the holo START domain on Golgi-like and neutral bilayers. Numbers are occupancies reported in percentage of simulation time.

Residues	Holo+Golgi_r1	Holo+Golgi_r2	Holo+Neu_r1	Holo+Neu_r2
PHE-436	0.02	0.04	0.00	0.00
ARG-442	0.18	0.19	0.15	2.33
GLU-446	0.39	2.45	2.46	4.29
GLN-467	8.41	0.31	0.37	28.08
HSD-469	0.02	0.01	0.74	0.01
VAL-472	0.00	0.00	1.77	0.00
TYR-482	1.56	16.16	6.02	3.58
ASN-504	31.71	47.06	20.97	28.43
TYR-553	93.11	94.12	58.36	95.14
GLU-575	0.00	0.00	0.24	0.00
TYR-576	3.72	3.89	7.99	2.69

Table S6. MSλD START-Cer relative binding free energy (ΔΔG) for the Y553F, N504A and V480A substitutions. The production simulations were run in five replicates (40 ns each).

Mutation	ΔG (kcal/mol)		$\Delta\Delta G_{binding}$ (kcal/mol)	Production run
	START-Cer-Water	START-Water	START-Cer	(ns)
Y553F	17.7 ± 0.1	16.9 ± 0.1	0.8 ± 0.1	200
N504A	81.5 ± 0.2	80.5 ± 0.2	1.1 ± 0.2	200
V480A	0.7 ± 0.1	-0.5 ± 0.1	1.2 ± 0.1	200

Table S7. MSλD START-Cer relative binding free energies for the V480A substitution in the presence of a POPC tail in the binding pocket. The production simulations were run in 5 replicates (30 ns each).

Mutation	ΔG (kcal/mol)		$\Delta\Delta G_{binding}$ (kcal/mol)	Production run
	START-Cer-POPC-Water	START-POPC-Water	START-Cer	(ns)
V480A	2.8 ± 0.3	2.7 ± 0.2	0.1 ± 0.2	150

Table S8: Phosphatidylcholine (PC) species observed with CERT and other known PC-binding lipid transfer proteins by LC-MS/MS. The different columns describe in order: the lipid transfer protein of interest; the PC species, the adduct as which the PC was observed, and the full identity of the PC with possible extra information on the fatty acyl composition; the MS intensity by which the PC was observed; its total carbon chain length and unsaturation; the MS ion mode in which the PC was observed and at what m/z and LC retention time range.

Protein	Lipid	Adduct	Full Identity Of Lipid	Intensity	Total Carbon Chain Length	Total Carbon Chain Unsaturations	Ion Mode	mz	Mean Of Retention Time	Minimum Of Retention Time	Maximum Of Retention Time
GM2A	PC(33:1)	[M+H]+	[M+H]+: Phosphatidylcholine (33:1)	470000	33	1	pos	746.5571	9.16	9.04	11.26
GM2A	PC(40:2)	[M+H]+	[M+H]+: Phosphatidylcholine (40:2)	420000	40	2	pos	842.6587	13.79	13.58	14.42
GM2A	PC(31:1)	[M+HCOO]-	[M+HCOO]-: Phosphatidylcholine (31:1) => (16:1/15:0)	92000	31	1	neg	762.5208	8.93	8.55	9.61
LCN1	PC(32:1)	[M+H]+	[M+H]+: Phosphatidylcholine (32:1)	5100000	32	1	pos	732.5501	9.34	8.58	10.19
LCN1	PC(36:2)	[M+H]+	[M+H]+: Phosphatidylcholine (36:2)	5000000	36	2	pos	786.5950	10.82	9.9	11.29
LCN1	PC(34:1)	[M+H]+	[M+H]+: Phosphatidylcholine (34:1)	4900000	34	1	pos	760.5771	10.66	10.36	11.09
LCN1	PC(34:2)	[M+H]+	[M+H]+: Phosphatidylcholine (34:2)	4000000	34	2	pos	758.5638	9.59	9.19	10.61
LCN1	PC(32:2)	[M+H]+	[M+H]+: Phosphatidylcholine (32:2)	2400000	32	2	pos	730.5339	8.48	8.00	9.81
LCN1	PC(30:1)	[M+H]+	[M+H]+: Phosphatidylcholine (30:1)	1800000	30	1	pos	704.5178	8.17	7.78	8.65
LCN1	PC(33:1)	[M+H]+	[M+H]+: Phosphatidylcholine (33:1)	660000	33	1	pos	746.5643	10.06	6.96	10.66
LCN1	PC(34:3)	[M+H]+	[M+H]+: Phosphatidylcholine (34:3)	520000	34	3	pos	756.5482	8.70	8.30	9.44
LCN1	PC(31:1)	[M+H]+	[M+H]+: Phosphatidylcholine (31:1)	440000	31	1	pos	718.5338	8.74	8.21	10.09
LCN1	PC(36:1)	[M+H]+	[M+H]+: Phosphatidylcholine (36:1)	380000	36	1	pos	788.6105	12.09	11.73	12.5
LCN1	PC(36:4)	[M+H]+	[M+H]+: Phosphatidylcholine (36:4)	280000	36	4	pos	782.5635	9.53	8.43	10.43
LCN1	PC(33:2)	[M+H]+	[M+H]+: Phosphatidylcholine (33:2)	260000	33	2	pos	744.5500	8.97	8.55	10.09
LCN1	PC(38:5)	[M+H]+	[M+H]+: Phosphatidylcholine (38:5)	230000	38	5	pos	808.5804	9.65	9.11	10.17

LCN1	PC(30:0)	[M+H]+	[M+H]+: Phosphatidylcholine (30:0)	230000	30	0	pos	706.5332	9.13	8.68	9.45
LCN1	PC(38:6)	[M+H]+	[M+H]+: Phosphatidylcholine (38:6)	210000	38	6	pos	806.5648	9.28	8.21	10.17
LCN1	PC(32:1)	[M+Na]+	[M+Na]+: Phosphatidylcholine (32:1)	150000	32	1	pos	754.5295	9.32	9.02	9.81
LCN1	PC(34:1)	[M+Na]+	[M+Na]+: Phosphatidylcholine (34:1)	140000	34	1	pos	782.5602	10.65	10.39	10.97
LCN1	PC(36:2)	[M+Na]+	[M+Na]+: Phosphatidylcholine (36:2)	140000	36	2	pos	808.5768	10.79	10.52	11.13
LCN1	PC(31:0)	[M+H]+	[M+H]+: Phosphatidylcholine (31:0)	120000	31	0	pos	720.5490	9.51	9.19	9.89
LCN1	PC(40:7)	[M+H]+	[M+H]+: Phosphatidylcholine (40:7)	120000	40	7	pos	832.5811	9.40	8.93	9.77
LCN1	PC(34:2)	[M+Na]+	[M+Na]+: Phosphatidylcholine (34:2)	110000	34	2	pos	780.5453	9.53	9.19	10.11
LCN1	PC(30:2)	[M+H]+	[M+H]+: Phosphatidylcholine (30:2)	130000	30	2	pos	702.5030	7.56	7.00	8.22
LCN1	PC(32:1)	[M+HCOO]-	[M+HCOO]-: Phosphatidylcholine (32:1) => (16:1/16:0)	140000	32	1	neg	776.5365	9.33	9.03	9.79
LCN1	PC(34:1)	[M+HCOO]-	[M+HCOO]-: Phosphatidylcholine (34:1) => (18:1/16:0)	110000	34	1	neg	804.5664	10.64	10.38	10.93
LCN1	PC(34:2)	[M+HCOO]-	[M+HCOO]-: Phosphatidylcholine (34:2) => (16:1/18:1)	100000	34	2	neg	802.5513	9.50	9.24	10.08
PITPNA	PC(33:1)	[M+H]+	[M+H]+: Phosphatidylcholine (33:1)	130000	33	1	pos	746.5789	9.68	9.14	10.54
PITPNA	PC(30:1)	[M+H]+	[M+H]+: Phosphatidylcholine (30:1)	120000	30	1	pos	704.5321	7.91	7.58	8.38
PITPNB	PC(32:1)	[M+H]+	[M+H]+: Phosphatidylcholine (32:1)	380000	32	1	pos	732.5632	9.14	7.14	10.41
PITPNB	PC(34:1)	[M+H]+	[M+H]+: Phosphatidylcholine (34:1)	340000	34	1	pos	760.5938	10.4	10.07	10.9
PITPNB	PC(34:2)	[M+H]+	[M+H]+: Phosphatidylcholine (34:2)	210000	34	2	pos	758.5788	9.40	9.00	10.73
PITPNB	PC(30:0)	[M+H]+	[M+H]+: Phosphatidylcholine (30:0)	33000	30	0	pos	706.5481	8.88	8.65	9.14
STARD2	PC(34:1)	[M+H]+	[M+H]+: Phosphatidylcholine (34:1)	17000000	34	1	pos	760.5780	10.98	10.72	11.41
STARD2	PC(36:2)	[M+H]+	[M+H]+: Phosphatidylcholine (36:2)	16000000	36	2	pos	786.5944	11.13	10.76	11.64
STARD2	PC(34:2)	[M+H]+	[M+H]+: Phosphatidylcholine (34:2)	11000000	34	2	pos	758.5645	9.94	9.49	10.98

STARD2	PC(32:1)	[M+H]+	[M+H]+: Phosphatidylcholine (32:1)	11000000	32	1	pos	732.5499	9.80	6.93	11.53
STARD2	PC(32:2)	[M+H]+	[M+H]+: Phosphatidylcholine (32:2)	3200000	32	2	pos	730.5345	8.74	8.34	9.50
STARD2	PC(36:3)	[M+H]+	[M+H]+: Phosphatidylcholine (36:3)	2700000	36	3	pos	784.5806	10.19	9.76	11.05
STARD2	PC(38:6)	[M+H]+	[M+H]+: Phosphatidylcholine (38:6)	1700000	38	6	pos	806.5657	9.64	8.47	10.21
STARD2	PC(38:5)	[M+H]+	[M+H]+: Phosphatidylcholine (38:5)	1700000	38	5	pos	808.5819	10.03	9.49	10.58
STARD2	PC(40:7)	[M+H]+	[M+H]+: Phosphatidylcholine (40:7)	1500000	40	7	pos	832.5814	9.79	9.49	10.12
STARD2	PC(36:4)	[M+H]+	[M+H]+: Phosphatidylcholine (36:4)	1600000	36	4	pos	782.5646	9.90	8.94	10.21
STARD2	PC(36:1)	[M+H]+	[M+H]+: Phosphatidylcholine (36:1)	1300000	36	1	pos	788.6122	12.41	12.15	12.83
STARD2	PC(30:1)	[M+H]+	[M+H]+: Phosphatidylcholine (30:1)	1200000	30	1	pos	704.5179	8.54	8.10	8.91
STARD2	PC(33:1)	[M+H]+	[M+H]+: Phosphatidylcholine (33:1)	1100000	33	1	pos	746.5662	10.29	9.28	10.85
STARD2	PC(34:3)	[M+H]+	[M+H]+: Phosphatidylcholine (34:3)	980000	34	3	pos	756.5484	9.09	8.60	9.96
STARD2	PC(38:3)	[M+H]+	[M+H]+: Phosphatidylcholine (38:3)	670000	38	3	pos	812.6126	11.77	10.39	12.42
STARD2	PC(38:7)	[M+H]+	[M+H]+: Phosphatidylcholine (38:7)	560000	38	7	pos	804.5499	8.56	8.26	9.90
STARD2	PC(34:1)	[M+Na]+	[M+Na]+: Phosphatidylcholine (34:1)	510000	34	1	pos	782.5605	10.97	10.76	11.28
STARD2	PC(38:4)	[M+H]+	[M+H]+: Phosphatidylcholine (38:4)	510000	38	4	pos	810.5961	11.27	10.81	11.59
STARD2	PC(36:5)	[M+H]+	[M+H]+: Phosphatidylcholine (36:5)	500000	36	5	pos	780.5490	8.80	8.04	9.43
STARD2	PC(40:6)	[M+H]+	[M+H]+: Phosphatidylcholine (40:6)	480000	40	6	pos	834.5973	10.15	9.66	10.83
STARD2	PC(35:1)	[M+H]+	[M+H]+: Phosphatidylcholine (35:1)	480000	35	1	pos	774.5956	11.55	11.08	11.91
STARD2	PC(40:6)	[M+H]+	[M+H]+: Phosphatidylcholine (40:6)	450000	40	6	pos	834.5957	11.02	10.76	11.38
STARD2	PC(36:2)	[M+Na]+	[M+Na]+: Phosphatidylcholine (36:2)	450000	36	2	pos	808.5762	11.12	10.89	11.6
STARD2	PC(34:2)	[M+Na]+	[M+Na]+: Phosphatidylcholine (34:2)	330000	34	2	pos	780.5457	9.89	9.54	10.46
STARD2	PC(31:1)	[M+H]+	[M+H]+: Phosphatidylcholine (31:1)	320000	31	1	pos	718.5341	9.08	8.52	9.62

STARD2	PC(32:1)	[M+Na]+	[M+Na]+: Phosphatidylcholine (32:1)	310000	32	1	pos	754.5300	9.79	9.37	10.16
STARD2	PC(31:0)	[M+H]+	[M+H]+: Phosphatidylcholine (31:0)	210000	31	0	pos	720.5496	9.82	9.58	10.21
STARD2	PC(30:0)	[M+H]+	[M+H]+: Phosphatidylcholine (30:0)	210000	30	0	pos	706.5342	9.38	9.12	9.72
STARD2	PC(36:6)	[M+H]+	[M+H]+: Phosphatidylcholine (36:6)	200000	36	6	pos	778.5341	8.35	7.73	9.49
STARD2	PC(34:1)	[M+HCOO]-	[M+HCOO]-: Phosphatidylcholine (34:1) => (18:1/16:0)	540000	34	1	neg	804.5667	10.92	10.66	11.27
STARD2	PC(36:2)	[M+HCOO]-	[M+HCOO]-: Phosphatidylcholine (36:2) => (18:1/18:1)	460000	36	2	neg	830.5823	11.09	10.8	11.56
STARD2	PC(32:1)	[M+HCOO]-	[M+HCOO]-: Phosphatidylcholine (32:1) => (16:1/16:0)	410000	32	1	neg	776.5366	9.61	9.28	10.13
STARD2	PC(34:2)	[M+HCOO]-	[M+HCOO]-: Phosphatidylcholine (34:2) => (18:1/16:1)	380000	34	2	neg	802.5512	9.80	9.48	10.38
STARD2	PC(32:2)	[M+HCOO]-	[M+HCOO]-: Phosphatidylcholine (32:2) => (16:1/16:1)	120000	32	2	neg	774.5207	8.69	8.28	9.32
STARD2	PC(34:1)	[M+HCOO+1x (HCOONa)]-	[M+HCOO+1x(HCOONa)]-: Phosphatidylcholine (34:1)	110000	34	1	neg	872.5539	10.92	10.67	11.22
STARD2	PC(36:2)	[M+HCOO+1x (HCOONa)]-	[M+HCOO+1x(HCOONa)]-: Phosphatidylcholine (36:2)	90000	36	2	neg	898.5689	11.07	10.8	11.52
STARD2	PC(34:1)	[M+HCOO+2x (HCOONa)]-	[M+HCOO+2x(HCOONa)]-: Phosphatidylcholine (34:1)	87000	34	1	neg	940.5403	10.92	10.66	11.22
STARD2	PC(32:1)	[M+HCOO+1x (HCOONa)]-	[M+HCOO+1x(HCOONa)]-: Phosphatidylcholine (32:1)	76000	32	1	neg	844.5222	9.68	9.32	10.04
STARD2	PC(34:2)	[M+HCOO+1x (HCOONa)]-	[M+HCOO+1x(HCOONa)]-: Phosphatidylcholine (34:2)	76000	34	2	neg	870.5377	9.80	9.53	10.3
STARD2	PC(34:1)	[M+HCOO+3x (HCOONa)]-	[M+HCOO+3x(HCOONa)]-: Phosphatidylcholine (34:1)	62000	34	1	neg	1008.5268	10.92	10.67	11.17
STARD10	PC(36:2)	[M+H]+	[M+H]+: Phosphatidylcholine (36:2)	11000000	36	2	pos	786.5987	10.68	10.29	11.04
STARD10	PC(34:2)	[M+H]+	[M+H]+: Phosphatidylcholine (34:2)	8700000	34	2	pos	758.5674	9.42	9.00	9.90
STARD10	PC(34:1)	[M+H]+	[M+H]+: Phosphatidylcholine (34:1)	4400000	34	1	pos	760.5822	10.53	10.2	10.85
STARD10	PC(32:1)	[M+H]+	[M+H]+: Phosphatidylcholine (32:1)	3800000	32	1	pos	732.5533	9.25	7.41	11.63
STARD10	PC(32:2)	[M+H]+	[M+H]+: Phosphatidylcholine (32:2)	1700000	32	2	pos	730.5380	8.29	7.83	9.48

STARD10	PC(36:3)	[M+H]+	[M+H]+: Phosphatidylcholine (36:3)	1600000	36	3	pos	784.5838	9.75	9.33	10.22
STARD10	PC(30:1)	[M+H]+	[M+H]+: Phosphatidylcholine (30:1)	720000	30	1	pos	704.5206	8.07	7.12	10.44
STARD10	PC(34:3)	[M+H]+	[M+H]+: Phosphatidylcholine (34:3)	680000	34	3	pos	756.5517	8.59	8.13	9.28
STARD10	PC(36:4)	[M+H]+	[M+H]+: Phosphatidylcholine (36:4)	570000	36	4	pos	782.5677	8.83	8.38	9.73
STARD10	PC(35:2)	[M+H]+	[M+H]+: Phosphatidylcholine (35:2)	450000	35	2	pos	772.5833	10.02	9.62	10.43
STARD10	PC(38:6)	[M+H]+	[M+H]+: Phosphatidylcholine (38:6)	400000	38	6	pos	806.5692	9.25	8.08	10.01
STARD10	PC(36:2)	[M+Na]+	[M+Na]+: Phosphatidylcholine (36:2)	350000	36	2	pos	808.5821	10.68	10.33	11.00
STARD10	PC(40:7)	[M+H]+	[M+H]+: Phosphatidylcholine (40:7)	320000	40	7	pos	832.5849	9.36	8.96	9.64
STARD10	PC(38:4)	[M+H]+	[M+H]+: Phosphatidylcholine (38:4)	290000	38	4	pos	810.6002	10.11	9.54	10.75
STARD10	PC(38:5)	[M+H]+	[M+H]+: Phosphatidylcholine (38:5)	270000	38	5	pos	808.5844	9.62	9.26	9.97
STARD10	PC(33:2)	[M+H]+	[M+H]+: Phosphatidylcholine (33:2)	190000	33	2	pos	744.5533	8.82	8.42	9.32
STARD10	PC(34:2)	[M+Na]+	[M+Na]+: Phosphatidylcholine (34:2)	170000	34	2	pos	780.5494	9.42	9.04	9.85
STARD10	PC(38:2)	[M+H]+	[M+H]+: Phosphatidylcholine (38:2)	160000	38	2	pos	814.6322	11.99	11.6	12.41
STARD10	PC(38:7)	[M+H]+	[M+H]+: Phosphatidylcholine (38:7)	140000	38	7	pos	804.5533	8.21	7.83	8.58
STARD10	PC(36:2)	[M+HCOO]-	[M+HCOO]-: Phosphatidylcholine (36:2) => (18:1/18:1)	270000	36	2	neg	830.5859	10.72	10.48	11.00
STARD10	PC(34:1)	[M+HCOO]-	[M+HCOO]-: Phosphatidylcholine (34:1) => (18:1/16:0)	100000	34	1	neg	804.5701	10.57	10.35	10.86
CERT	PC(34:1)	[M+H]+	[M+H]+: Phosphatidylcholine (34:1)	610000	34	1	pos	760.5778	10.31	9.97	11.14
CERT	PC(32:1)	[M+H]+	[M+H]+: Phosphatidylcholine (32:1)	250000	32	1	pos	732.5490	9.03	6.24	10.66

Movie S1. POPC lipid inserts a tail in the cavity in the simulation of the apo+ER_r1. Simulation excerpt is from 400-950ns. POPC lipid is shown in purple. Ω1 loop colored orange, Ω4 loop colored cyan and START domain colored grey. Membrane lipid molecules are shown in licorice representation and for the sake of clarity, water molecules are not shown and only a subset of the lipids are shown.

Movie S2A. POPC inserts a tail in the cavity in the simulation of the holo+Neu_r1. Simulation excerpt is from 800-950ns. Ceramide lipid is shown in yellow, POPC lipid is shown in purple. Ω1 loop colored orange, Ω4 loop colored cyan and START domain colored grey. Membrane lipid molecules are shown in licorice representation and for the sake of clarity, water molecules are not shown and only a subset of the lipids are shown.

Movie S2B. Release of the bound ceramide from the cavity into the bilayer facilitated by presence of POPC tail in the cavity in the simulation of the holo+Neu_r1. Simulation excerpt is from 1380-1550ns. Ceramide lipid is shown in yellow, POPC lipid is shown in purple. Ω1 loop colored orange, Ω4 loop colored cyan and START domain colored grey. Membrane lipid molecules are shown in licorice representation and for the sake of clarity, water molecules are not shown and only a subset of the lipids are shown.

Movie S2C. Released ceramide returns to the cavity when POPC tails exits the cavity in the simulation of the holo+Neu_r1. Simulation excerpt is from 1570-1770ns. Ceramide lipid is shown in yellow, POPC lipid is shown in purple. Ω1 loop colored orange, Ω4 loop colored cyan and START domain colored grey. Membrane lipid molecules are shown in licorice representation and for the sake of clarity, water molecules are not shown and only a subset of the lipids are shown.

Movie S3. POPC inserts a tail in the cavity in the simulation of the holo+Golgi_r1. Simulation excerpt is from 810-940ns. Ceramide lipid is shown in yellow, POPC lipid is shown in purple. Ω1 loop colored orange, Ω4 loop colored cyan and START domain colored grey. Membrane lipid molecules are shown in licorice representation and for the sake of clarity, water molecules are not shown and only a subset of the lipids are shown.

Movie S4. Release of the ceramide from the cavity followed by uptake of POGL into the cavity in the simulation of the Holo+Golgi+POPC_r1. Simulation excerpt is from 100-1500ns. Ceramide lipid is shown in yellow, POPC lipid is shown in purple, POGL lipid is shown in magenta. Ω1 loop colored orange, Ω4 loop colored cyan and START domain colored grey. Membrane lipid molecules are shown in licorice representation and for the sake of clarity, water molecules are not shown and only a subset of the lipids are shown.

References:

(1) Feig, M.; Karanicolas, J.; Brooks, C. L. MMTSB Tool Set: Enhanced Sampling and Multiscale Modeling Methods for Applications in Structural Biology. *J. Mol. Graph. Model.* **2004**, *22*, 377–395. https://doi.org/10.1016/j.jmgm.2003.12.005.

(2) Brooks, B. R.; Brooks, C. L.; Mackerell, A. D.; Nilsson, L.; Petrella, R. J.; Roux, B.; Won, Y.; Archontis, G.; Bartels, C.; Boresch, S.; Caflisch, A.; Caves, L.; Cui, Q.; Dinner, A. R.; Feig, M.; Fischer, S.; Gao, J.; Hodoscek, M.; Im, W.; Kuczera, K.; Lazaridis, T.; Ma, J.; Ovchinnikov, V.; Paci, E.; Pastor, R. W.; Post, C. B.; Pu, J. Z.; Schaefer, M.; Tidor, B.; Venable, R. M.; Woodcock, H. L.; Wu, X.; Yang, W.; York, D. M.; Karplus, M. CHARMM: The Biomolecular Simulation Program. *J. Comput. Chem.* **2009**, *30*, 1545–1614. https://doi.org/10.1002/jcc.21287.

(3) Ryckaert, J.-P.; Ciccotti, G.; Berendsen, H. J. C. Numerical Integration of the Cartesian Equations of Motion of a System with Constraints: Molecular Dynamics of n-Alkanes. *J. Comput. Phys.* **1977**, *23*, 327–341. https://doi.org/10.1016/0021-9991(77)90098-5.

(4) Hayes, R. L.; Buckner, J.; Brooks, C. L. BLaDE: A Basic Lambda Dynamics Engine for GPU-Accelerated Molecular Dynamics Free Energy Calculations. *J. Chem. Theory Comput.* **2021**, *17*, 6799–6807. https://doi.org/10.1021/acs.jctc.1c00833.

(5) Tubiana, T.; Carvaillo, J.-C.; Boulard, Y.; Bressanelli, S. TTClust: A Versatile Molecular Simulation Trajectory Clustering Program with Graphical Summaries. *J. Chem. Inf. Model.* **2018**, *58*, 2178–2182. https://doi.org/10.1021/acs.jcim.8b00512.

(6) Hayes, R. L.; Vilseck, J. Z.; Brooks, C. L. Approaching Protein Design with Multisite λ Dynamics: Accurate and Scalable Mutational Folding Free Energies in T4 Lysozyme. *Protein Sci.* **2018**, *27*, 1910–1922. https://doi.org/10.1002/pro.3500.

(7) Hayes, R. L.; Brooks, C. L. A Strategy for Proline and Glycine Mutations to Proteins with Alchemical Free Energy Calculations. *J. Comput. Chem.* **2021**, *42*, 1088–1094. https://doi.org/10.1002/jcc.26525.

(8) Hayes, R. L.; Armacost, K. A.; Vilseck, J. Z.; Brooks, C. L. Adaptive Landscape Flattening Accelerates Sampling of Alchemical Space in Multisite λ Dynamics. *J. Phys. Chem. B* **2017**, *121*, 3626–3635. https://doi.org/10.1021/acs.jpcb.6b09656.

(9) Hynninen, A.; Crowley, M. F. New Faster CHARMM Molecular Dynamics Engine. *J. Comput. Chem.* **2014**, *35*, 406–413. https://doi.org/10.1002/jcc.23501.

(10) Kong, X.; Brooks, C. L. λ-Dynamics: A New Approach to Free Energy Calculations. *J. Chem. Phys.* **1996**, *105*, 2414–2423. https://doi.org/10.1063/1.472109.

(11) Knight, J. L.; Brooks, C. L. Multisite λ Dynamics for Simulated Structure–Activity Relationship Studies. *J. Chem. Theory Comput.* **2011**, *7*, 2728–2739. https://doi.org/10.1021/ct200444f.

(12) Kumar, S.; Rosenberg, J. M.; Bouzida, D.; Swendsen, R. H.; Kollman, P. A. THE Weighted Histogram Analysis Method for Free-Energy Calculations on

Biomolecules. I. The Method. *J. Comput. Chem.* **1992**, *13*, 1011–1021. https://doi.org/10.1002/jcc.540130812.
